# Open- and Closed-Skill Exercise Interventions Produce Different Neurocognitive Effects on Executive Functions in the Elderly: A 6-Month Randomized, Controlled Trial

**DOI:** 10.3389/fnagi.2017.00294

**Published:** 2017-09-12

**Authors:** Chia-Liang Tsai, Chien-Yu Pan, Fu-Chen Chen, Yu-Ting Tseng

**Affiliations:** ^1^Institute of Physical Education, Health and Leisure Studies, National Cheng Kung University Tainan, Taiwan; ^2^Department of Physical Education, National Kaohsiung Normal University Kaohsiung, Taiwan; ^3^School of Kinesiology, University of Minnesota Minneapolis, MN, United States

**Keywords:** cognition, behavior, event-related potential, exercise modes, elderly

## Abstract

This study aimed to explore the effects of open- and closed-skill exercise interventions on the neurocognitive performance of executive functions in the elderly. Sixty-four healthy elderly males were randomly assigned to either a closed-skill (bike riding or brisk walking/jogging, *n* = 22), open-skill (table tennis, *n* = 21), or control (*n* = 21) group. Various neuropsychological [e.g., accuracy rates (AR) and reaction time (RT)] and electrophysiological [e.g., event-related potential (ERP) P3 component] measures were assessed during a variant of the task-switching paradigm, as well as an N-back task at baseline and after either a 6-month exercise intervention or control period. The results showed that, when performing the task-switching paradigm, the two exercise groups relative to control group showed significantly faster RTs in the switch trials after the exercise intervention. However, the RT facilitation in the non-switch and switch trials post-exercise relative to pre-exercise only emerged in the open-skill group. In terms of the N-back task, the two exercise groups significantly increased ARs in the 1-back condition after the exercise intervention, and the beneficial AR effect on the 2-back condition only emerged in the closed-skill group. In addition, the two exercise groups exhibited significantly larger P3 amplitudes on the frontal-to-parietal cortex areas after the exercise intervention relative to the baseline when performing the two cognitive tasks. These neurocognitive results still remained unchanged even when the confounding factors (e.g., cardiorespiratory fitness, social participation, and BMI) were controlled for. The present study concluded that, although 6-month open- and closed-skill exercise interventions facilitate overall electrophysiological effects (i.e., increased ERP P3 amplitudes) on the frontal-to-parietal cortices in the elderly, the two exercise modes produced different levels of neuropsychologically beneficial effects on RTs of the task-switching paradigm (i.e., lessened RTs) and ARs of the N-back task (i.e., enhanced ARs). The distinctive neurocognitive changes induced by open- and closed-skill exercise have implications for task switching and working memory in elderly individuals, especially with such cognitive functioning impairments.

## Introduction

Life expectancy has been increasing in developed countries, resulting in a rapid growth in the elderly population. Since aging is the main risk factor for neurodegenerative diseases (e.g., Alzheimer's disease), which affect a substantial and growing part of the global population (Rodríguez-Arellano et al., 2016), one important issue is how to counteract such neurocognitive declines in order to reduce the medical costs associated with geriatric care. Although a decrease in certain cognitive functions is an unavoidable part of normal aging, the degree to which this occurs varies within the healthy older population. Among an array of cognitive functions, the executive-control processes and brain areas that support them have been shown to undergo large age-related performance declines (West, 1996; Colcombe and Kramer, 2003; Anderson and McConnell, 2007), as seen in capacities such as working memory (Wingfield et al., 1988), visuospatial attention (Greenwood et al., 1993), and task switching (Friedman et al., 2008).

The vast majority of related studies have proposed that physical fitness and exercise are factors that strongly promote healthy cognitive aging (Lee et al., 2012; Kimura et al., 2013; Tsai et al., 2015). Improvements in physical fitness via exercise training are thus reflected in enhancements of a number of cognitive functions, such as processing speed, visuospatial function, and control processes (e.g., inhibition, planning, scheduling, and working memory) in older adults, with the largest effect sizes being on tests thought to depend more on the executive function (e.g., task switching, inhibitory control, and working memory; Diamond, 2013) (Hall et al., 2001; Voelcker-Rehage et al., 2010; Guiney and Machado, 2013; Tsai et al., 2015). However, a broad range of physical exercise types is possible, and different kinds of exercise seem to have specific effects on neurocognitive performance, due to the differences in the secretion of some biomarkers (e.g., brain-derived neurotrophic factor, insulin-like growth factor-1, and homocysteine) in the neurochemical system (Neeper et al., 1995; Liu-Ambrose et al., 2010; Cassilhas et al., 2012; Tsai et al., 2014b,c), and the differences in brain tissue volumes and activation patterns induced by different types of exercise (Luft et al., 2008; Park et al., 2008; Liu-Ambrose et al., 2010; Erickson et al., 2011; Tsai and Wang, 2015; Tsai et al., 2016). These earlier works seem to support the view that different types of physical exercise could affect the brain in different ways.

Different forms of physical exercise with different cognitive executive process loads and different motor-coordination skills have been reported to be strongly associated with improved neurocognitive performances (Voelcker-Rehage et al., 2011; Tsai et al., 2016). Therefore, the present study divided exercise into two main modes, open- and closed-skill (Di Russo et al., 2010; Dai et al., 2013; Tsai and Wang, 2015), since the former (e.g., table tennis and badminton) requires rich cognitive and executive loadings and different sets of motor-coordination skills to adapt to a unpredictable/changing environment and various opponents (van Praag et al., 2000; Artola et al., 2006; Di Russo et al., 2010), while the later (e.g., running and biking) is performed according to the individual's own pace in a stable and predictable environment (Di Russo et al., 2010). From the perspective of motor-coordination skill, Voelcker-Rehage et al. (2011) found that older adults experienced beneficial effects on executive control (assessed using the Flanker task) and perceptual speed (assessed using the Visual Search task) due to cardiorespiratory and coordination training. However, the two exercise modes produced different effects on speed and accuracy, with coordination training leading to improved accuracy rates (ARs) on executive control and perceptual speed, but cardiorespiratory training only leading to better ARs on executive control. In terms of reaction time (RT), only the perceptual speed task was significantly improved by coordination training. In addition, cardiorespiratory training increased activation of the sensorimotor network in the elderly, while coordination training elevated activation of the visual–spatial network (Voelcker-Rehage et al., 2011). In contrast, Hötting et al. (2012) found that, although significant increases in episodic memory learning scores were found for both the cycling and stretching/coordination groups as compared with the sedentary control group, cycling training had greater effects on the episodic memory recognition scores than the stretching/coordination training. They also found that the latter was particularly effective in improving selective attention as compared with the cycling training. This suggests a specific relation between particular types of exercise and cognitive functions, with the increase in memory functions being linked to an increase in cardiovascular fitness, whereas the increase in attention is more pronounced after stretching/coordination training. The two exercise modes (i.e., open- and closed-skills) thus seem to be capable of producing different effects on the various cognitive domains (Voss et al., 2010) and neural processes (Tsai and Wang, 2015; Tsai et al., 2016).

Although recent studies have explored the effects of open- and closed-skill exercise on neuropsychological performance in disabled athletes (Di Russo et al., 2010) and the young adults (Wang et al., 2013a,b), and neurocognitive (neuropsychological and electrophysiological) performances in the elderly (Dai et al., 2013; Tsai and Wang, 2015; Tsai et al., 2016), the findings in the rather limited research literature remain somewhat ambiguous. More importantly, even though previous cross-sectional studies have demonstrated that regular participation in open- and closed-skill exercise has distinct benefits for neurocognitive performances (e.g., specific cost, RT, P3 amplitudes, and strength of inhibitory control) in the elderly when performing the task switching paradigm (Dai et al., 2013; Tsai and Wang, 2015) and visuospatial attention task (Tsai et al., 2016), the elderly subjects who showed more of the benefits of open-skill exercise on neurocognitive performance might have had some inherently better aspects of their executive control functions (e.g., visuospatial attention and task switching) as compared to their counterparts participating in the closed-skill exercise mode, and this may have induced them to adopt this kind of exercise (Snowden et al., 2011). Therefore, these cross-sectional studies cannot establish causality between exercise and cognitive aging, which is nonetheless required for more accurate and effective public health recommendations (Snowden et al., 2011; Miller et al., 2012), as well as to better explain the beneficial effects of the two exercise modes.

Two cognitive tasks, the task-switch paradigm and N-back task, were adopted in the current study to investigate the impacts of the various exercise-mode mechanisms responsible for specific kinds of executive-control functioning, since earlier works found that open- and closed-skill exercise could have different neurocognitive effects on different cognitive tasks executive functions (Di Russo et al., 2010; Dai et al., 2013; Tsai and Wang, 2015; Tsai et al., 2016), and, crucially, open-skill exercise (e.g., table tennis) affects more of the prefrontal cortex areas responsible for attention, task-switching and inhibition, while closed-skill exercise (e.g., jogging) works more on the hippocampus, which is important for memory (e.g., long-term memory and working memory; Axmacher et al., 2010; Burrel, 2015). Moreover, there are previous reports of age- and physical-activity-related impacts on the neuropsychological (e.g., AR and RT) and electrophysiological [e.g., event-related potential (ERP) P3 component] outcomes of the task-switching paradigm (Hillman et al., 2006; Friedman et al., 2008; Adrover-Roig and Barceló, 2010; Guiney and Machado, 2013) and N-back task (Voelcker-Rehage et al., 2010; Guiney and Machado, 2013; Saliasi et al., 2013) among elderly subjects.

ERP recordings made during the cognitive task performance permitted on-line measures of cognitive processes on the order of milliseconds, which cannot be obtained by neuropsychological performance alone (Tsai et al., 2014b). Given that the P3 activity has nonspecific qualities that are often associated with indexing stimulus evaluations and the intensity of the concomitant executive function processes (e.g., task-set updating processes and reconfiguration, updating working memory, integrating information into existing networks) (Kok, 2001; Kieffaber and Hetrick, 2005; Nicholson et al., 2005; Polich, 2007), the ERP component was used to illustrate the effects of the different exercise-mode interventions on executive cognitive functions in the elderly in the present study. With regard to the electrophysiological index, the P3 activity induced by the task-switch paradigm represents the set of processes subsumed under the construct of the task-set reconfiguration (Kieffaber and Hetrick, 2005; Nicholson et al., 2005). ERP P3 represents the memory-related neural processing that is involved in categorizing incoming information and updating the context of the working memory (e.g., encoding, rehearsal, recognition, and retrieval) (Duncan-Johnson and Donchin, 1977; Donchin and Coles, 1988; Rugg, 1995).

To date there is a lack of intervention research on the impact of open- and closed-skill exercise modes on various forms of executive function (e.g., task switching and working memory) involved in cognitive aging in older adults. The main goal of this study was thus to clarify the distinctive effects of a 6-month open- and closed-skill exercise intervention on the neuropsychological (e.g., AR and RT) and electrophysiological (e.g., ERP P3 latency and amplitude) performances in older adults with a sedentary life-style when performing the task-switching paradigm and N-back task, with rigorous controls on the confounding factors in neurocognitive performance [e.g., cardiorespiratory fitness, social participation, and body mass index (BMI), since these parameters could be changed to different extents after exercise] (Messier and Gagnon, 2009; Miller et al., 2012). The elderly subjects with regular open-skill exercise participation in the literature general show better switch-related neurocognitive performances than those with only closed-skill experience (Dai et al., 2013; Tsai and Wang, 2015), and short-term closed-skill exercise intervention (e.g., resistance exercise) cannot improve task-switching performance (Kimura et al., 2010), while closed-skill exercise training with the goal of enhancing cardiorespiratory fitness can facilitate white matter integrity, increase the size of the hippocampus, and improve memory performance in the elderly (Erickson et al., 2011; Ruscheweyh et al., 2011; Voss et al., 2013; Maass et al., 2016) and have more benefits on memory functions than stretching/coordination training (Hötting et al., 2012). As such, we hypothesized the following: (1) that a 6-month open-skill exercise intervention (e.g., table tennis) in contrast to a closed-exercise one (e.g., bike riding or brisk walking/jogging) would have more benefits for neurocognitive performance with regard to task-switching in the elderly; and (2) that closed-skill exercise would have more beneficial effects on the cognitive functioning involving the memory domains.

## Materials and methods

### Participants

Sixty-four community-dwelling men were recruited with the use of an informative flyer and underwent screening by a standardized telephone interview, with subjects being eligible for inclusion in this study if they (1) were aged 60–80 years old and a non-smoker; (2) were living independently in their own home with a sedentary life-style; and (3) did not have a current medical condition for which exercise is contraindicated. The participants consisted solely of men because neurocognitive and endocrinological responses to exercise could be gender-dependent (Baker et al., 2010). They then underwent a routine laboratory testing and medical examination, including blood pressure and heart rate measurements, electrocardiography, a standardized neurological and psychiatric examination, and a structured interview on previous medical history to ascertain whether they were free of a history of neurological disorders, brain injury, depressive symptoms [scores above 13 on the Beck Depression Inventory, 2nd edition (BDI-II)], and cognitive impairment [scores below 26 on the Mini Mental State Examination (MMSE)] (Ruscheweyh et al., 2011). The Edinburgh Handedness Inventory assessed all participants as right-handed (Oldfield, 1971). Written informed consent, as approved by the Institutional Ethics Committees in the organization within which the study was performed, was obtained from all the participants.

### Procedures

**Figure 1:**
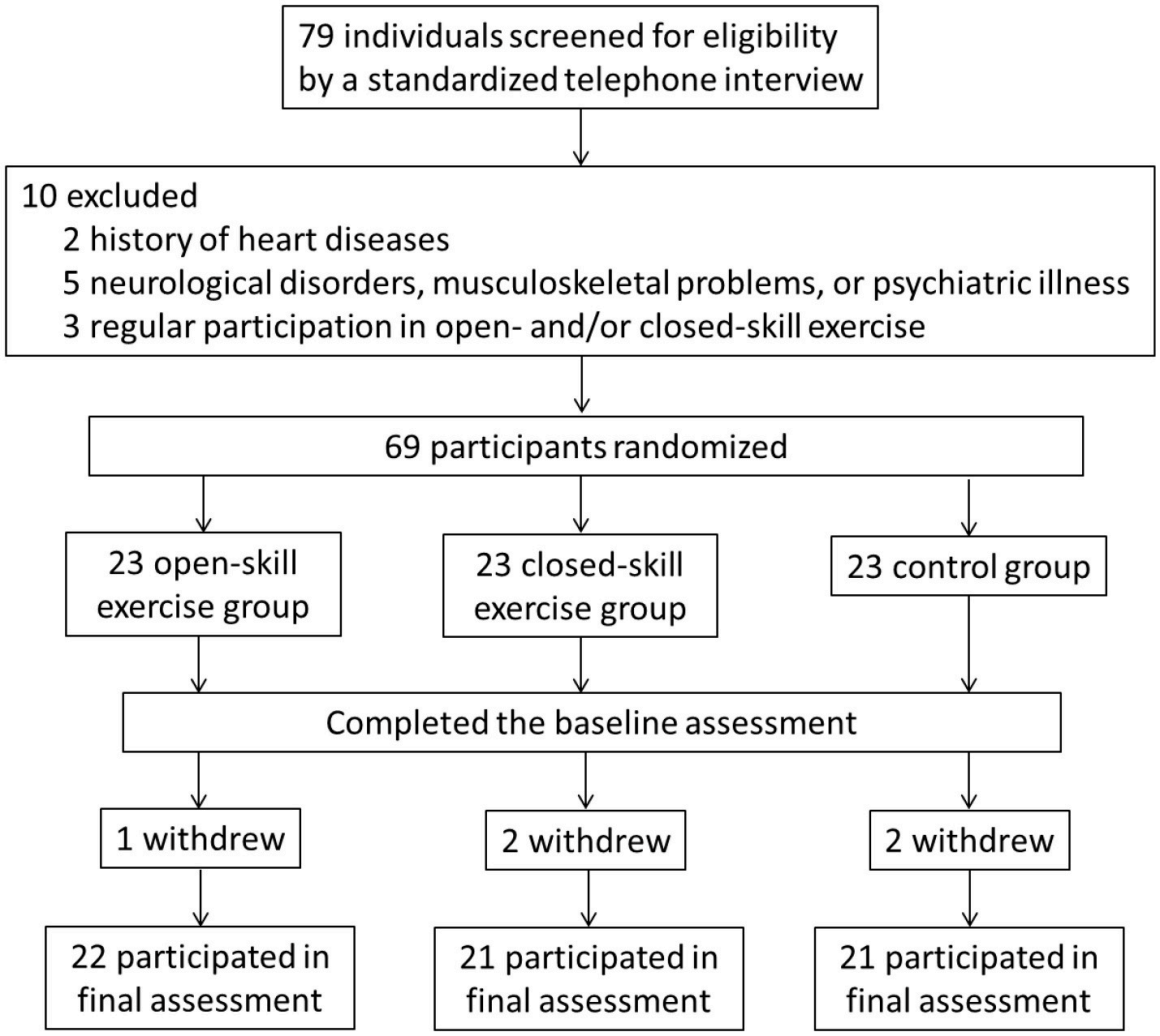
The CONSORT (Consolidated Standards of Reporting Trials) flowchart.

The Consolidated Standards of Reporting Trials (CONSORT) flowchart outlining the number of participants from first contact to study completion is shown in Figure 1. The original cohort consisted of 79 participants. After the assessment of a physician specializing in geriatric care, two subjects were excluded due to a history of heart disease, five due to neurological disorders, musculoskeletal problems, or psychiatric illness (e.g., scores above 13 on the BDI-II or below 26 on the MMSE), and three due to regular participation in open- and/or closed-skill exercise in the previous 3 months. The remaining 69 participants were randomly assigned to either an open-skill, closed-skill, or active control group by drawing an envelope with the treatment assignment enclosed after matching for age.

Before the exercise intervention, the participants visited the cognitive neurophysiology laboratory on two separate occasions. During the first session, each participant completed an informed consent form, the basic information form (e.g., a medical history and demographic questionnaire), and a handedness inventory. Two certified fitness instructors then completed all assessments of senior functional physical fitness for each participant. On a separate day within 1 week of the completion of the baseline evaluation, the participants performed two cognitive tasks (i.e., a task-switching paradigm and an N-back task) in a counter balanced order with concomitant electrophysiological recording (i.e., event-related potentials, ERPs).

Before the final exercise interventions were completed, one participant in the open-skill group, two in the closed-skill group, and two in the active control group, terminated their participation, leaving 64 participants in the final sample (open-skill, *n* = 22; closed-skill, *n* = 21; active control, *n* = 21). The three groups did not significantly differ at baseline in any of the demographic characteristics, including years of formal education, body mass index, systolic and diastolic pressure, social participation, MMSE, BDI-II, and senior functional physical fitness (see Table 1). Within 1 week after completing a 24-week exercise intervention, the participants completed the same questionnaires and senior functional physical fitness assessment, and received the same neurocognitive measurements, as in the pre-exercise procedure. Compliance with the prescribed training protocol remained high throughout the study period (90 ± 2%).

**Table 1:** Demographic characteristics of the open-skill exercise, closed-skill and control groups before and after the exercise intervention.

	**Open-skill (*n* = 22)**			**Closed-skill (*n* = 21)**			**Control (*n* = 21)**		
	**Pre-exercise**	**Post-exercise**	***p***	**Pre-exercise**	**Post-exercise**	***p***	**Pre-exercise**	**Post-exercise**	***p***
Age (years)	66.88 ± 4.74		–	66.15 ± 4.90		–	65.70 ± 3.54		–
Education (years)	12.50 ± 4.09		–	12.62 ± 2.97		–	10.62 ± 3.20		–
Height (cm)	162.90 ± 6.71	162.88 ± 6.74	0.329	159.70 ± 7.24	159.57 ± 7.12	0.186	161.20 ± 7.94	160.68 ± 8.54	0.329
Weight (kg)	62.65 ± 9.11	62.71 ± 8.99	0.329	60.98 ± 9.11	58.86 ± 9.55	0.005	62.04 ± 10.57	61.95 ± 10.47	0.329
BMI (kg/m^2^)	23.65 ± 3.64	23.68 ± 3.61	0.329	23.79 ± 3.65	23.04 ± 2.99	0.007	23.83 ± 3.29	23.94 ± 3.20	0.458
Systolic pressure (mmHg)	128.68 ± 22.95	125.82 ± 18.02	0.076	129.29 ± 21.79	124.57 ± 20.99	0.125	132.05 ± 25.12	129.33 ± 24.59	0.313
Diastolic pressure (mmHg)	75.86 ± 11.62	74.14 ± 9.13	0.296	78.62 ± 11.65	74.76 ± 10.76	0.151	79.48 ± 10.58	77.67 ± 12.07	0.347
Social participation	10.23 ± 2.07	10.73 ± 1.91	0.031^*^	9.57 ± 2.40	9.90 ± 2.28	0.090	9.95 ± 2.04	9.62 ± 1.99	0.090
Memory depth	18.50 ± 5.57	19.45 ± 5.41	0.404	17.10 ± 7.15	20.14 ± 4.60	0.059	18.24 ± 3.89	18.67 ± 4.46	0.410
MMSE	28.73 ± 1.28	28.05 ± 2.73	0.252	27.48 ± 3.03	28.95 ± 1.40	0.082	27.67 ± 1.80	28.19 ± 1.43	0.299
BDI-II	3.68 ± 3.72	3.09 ± 3.05	0.221	5.04 ± 4.14	4.71 ± 8.16	0.877	3.38 ± 3.38	2.47 ± 3.03	0.092
**FITNESS**									
Grip (kg)	28.90 ± 6.75	29.13 ± 6.74	0.834	26.98 ± 10.03	24.65 ± 9.50	0.368	27.81 ± 7.62	26.84 ± 6.82	0.338
Arm Curl (number)	18.09 ± 4.60	21.82 ± 3.40	<0.001^*^	17.24 ± 3.22	17.29 ± 2.57	0.942	17.47 ± 4.32	16.95 ± 3.69	0.451
Chair Stand (sec)	15.18 ± 3.80	19.41 ± 5.90	0.001^*^	18.08 ± 6.35	17.95 ± 4.04	0.932	15.80 ± 4.98	16.10 ± 2.96	0.757
8-Foot Up-and-Go (sec)	6.03 ± 1.38	5.53 ± 1.03	0.012^*^	6.97 ± 3.73	5.54 ± 1.04	0.086	6.32 ± 1.09	6.35 ± 1.05	0.859
Back Scratch (cm)	−3.23 ± 11.98	−2.11 ± 9.66	0.315	−1.43 ± 11.14	−1.55 ± 9.37	0.924	−0.64 ± 7.31	0.43 ± 9.58	0.383
Chair Sit-and-Reach (cm)	19.85 ± 5.27	20.40 ± 3.06	0.532	17.09 ± 2.02	17.04 ± 2.16	0.782	18.58 ± 2.38	19.33 ± 2.84	0.181
VO_2max_ (mL/kg/min)	25.63 ± 9.66	30.32 ± 12.03	0.071	26.83 ± 5.44	31.01 ± 4.25	0.001^*^	24.11 ± 6.61	23.79 ± 6.71	0.735

*BMI, body mass index; MMSE, Mini Mental State Examination; BDI-II, Beck Depression Inventory, 2nd edition; VO_2max_, maximal oxygen uptake*.

* *p < 0.05*.

### Training protocol

#### Open-skill exercise condition

The participants in the open-skill group were trained individually and regularly in a series of 40-min sessions that took place three times per week for 24 weeks, with the following structure: a warm-up, the main part of table tennis training, playing games of table tennis with the coach, and cooling down at the end. The training intervention was carried out in a sequence of increasing complexity. The table tennis training program was intended to improve the participants' general skills, and had seven main components over the whole training session: (a) footwork (e.g., ready position, one-, two-, and cross-step), (b) serving (including how to give sidespin, backspin, topspin, no-spin, and so on), (c) forehand and backhand driving, (d) forehand bouncing, backhand bouncing, and alternate bouncing, (e) smashing, (f) continuously hitting back a ball that was randomly delivered by the ball-projection machine from fixed or random directions, and (g) comprehensive practice. Training for each skill began with a simple movement and then progressed to more complex variations. The assessor for the neurocognitive tests and the exercise leader/coach were blinded to group membership. A more detailed training manual is available on request from the authors.

#### Closed-skill exercise condition

Participants in the closed-skill group attended three supervised exercise sessions per week for 24 consecutive weeks on a bicycle ergometer or a motor-driven treadmill (Medtrack ST55, Quinton Instrument Company, United States), with exercise intensity corresponding to 50–60% of the individual target heart rate reserve (HRR) during the first 2 weeks and 70–75% of the HRR for the remainder of the program. Each aerobic exercise session involved a 5-min warm-up period, followed by 30 min of continuous bike riding or brisk walking/jogging at an intensity that would maintain the heart rate within the assigned training range, and 5 min of cool-down. A Polar HR monitor (RX800CX, Finland) was used to monitor each participant's heart rate during the exercise.

#### Control condition

Participants in the active control group attended a balance and stretching program led by a trained exercise leader three times a week for 24 weeks. Every class included a 5-min warm-up period, static stretching and balance training, and a 5-min cool-down period. Different stretching and balancing techniques used various equipment, such as balance boards and fitness balls, to maintain the participants' interest.

### Physical fitness assessment

The Senior Functional Physical Fitness (SFPF) test (Rikli and Jones, 2012) is a battery of tests that was used to assess the participants' physical fitness in the current study. The participants first undertook 10 min' warm-up before the test and then completed the component tests in the designated order. Five items in the SFPF test were measured, as follows: (1) the Arm Curl test, which assesses arm muscle (specifically of the biceps) strength endurance, with the score being the number of repetitions in 30 s using the elbow of the dominant hand to flex and extend with a weight (men: 8 lb; women: 5 lb) through the complete range of motion; (2) the Chair Stand test, which measures lower body strength, based on the number of repetitions in 30 s using a full standing position from a chair and then returning to a fully seated position; (3) the 8-Foot Up-and-Go test, which evaluates agility and dynamic balance, using the number of seconds needed to get up from a seated position from a chair, walk eight feet, and return to a fully seated position on the chair; (4) the Back Scratch test, which assess upper body (shoulder) flexibility based on the number of centimeters being short of touching (minus score) or overlapping (plus score) between the third fingertip of each hand; (5) the Chair Sit-and-Reach test, which assesses the flexibility of the lower extremities, with the score being the distance in centimeters between the fingertips and toes; the number of centimeters short of reaching the toes (minus score) or reached beyond the toes (plus score). With regard to cardiorespiratory fitness, the Rockport Fitness Walking Test was used to estimate VO_2max_ (Kline et al., 1987), in which the participants were required to walk one mile as quickly as possible, during which their heart rate was continuously recorded using a Polar HR monitor.

### Cognitive tasks

#### Task-switching paradigm

The task switching paradigm employed in the present study has been shown to effectively assess the variations among elderly subjects who regularly participate in open- and closed-skill types of exercise (Tsai and Wang, 2015). The stimulus used in this test was a white digit (1–9, excluding 5) shown focally in the center of the screen against a black background. The same digit was not repeated in successive trials, and the digits were put into eight task blocks (blocks 1–2 and 7–8: homogeneous tasks; blocks 3–6: heterogeneous tasks), with a short rest period in the middle of each. The homogenous (i.e., non-switch) blocks each included 56 trials. Within each homogenous block, the participants only responded if the focal digit was greater or less than 5 (e.g., blocks 1 and 7), or if it was odd or even (e.g., blocks 2 and 8). The heterogeneous (i.e., task-switching) blocks contained 224 trials, each with 20 switches. With the heterogeneous blocks, the participants started with one task (e.g., even/odd) and then switched to the other (e.g., more/less than 5), as signaled by a simultaneously presented rectangle drawn around the digit, after at least seven or no more than 13 intervening trials. The participants were asked to press one of two buttons on a small response box that they held in the right hand as quickly and accurately as possible. The digit was shown on the screen until the participants pressed the response button, and the following trial began 500ms after the RT response. The prompts “more less” or “even odd” appeared simultaneously with and below the digit during all trials, dependent on which was appropriate to the task. The homogenous and heterogeneous blocks were counterbalanced across participants. All the participants were given instructions about the tasks, and both single-task and task-switch trials were practiced before the formal test until they understood the whole procedure.

#### N-back task

A continuous stream of white letters (stimuli) with pseudo-random sequences of vowels and consonants was presented with 10% gray noise, embedded in a 50% random noise gray rectangular background patch, on a computer screen. Targets were defined according to the N-back design. Participants pressed a button with the index finger of their right hands as soon as a target appeared, and no motor response was needed for non-target trials. Stimuli were presented for a duration of 500 ms, followed by a 3 s inter-trial-interval during which a dot helped participants maintain fixation. The cognitive task consisted of three different working-memory-load conditions: (1) a simple detection (control) condition with sequential letters or background patches without any letters being presented, during which the participants responded when the background patches without letters appeared (memory-free condition); (2) the 1-back condition, with the target being any letter identical to the one immediately preceding it (moderately demanding); (3) the 2-back condition, with the target being any letter that was identical to the one presented two trials back (highly demanding). Before the formal test, the participants were given the task instructions and initially practiced a brief version of the task, consisting of two blocks of 45 trials each (one block of moderately demanding and one of highly demanding trials). Following this practice, the participants completed nine blocks of trials with 120 trials in each (three blocks per condition). Nine blocks were tested following the sequence: blocks 1, and 8–9: the control condition, blocks 2, and 6–7: the 1-back condition, and blocks 3–5: the 2-back condition. No feedback was provided during this period.

### ERPs recording and analysis

Continuous electroencephalographic (EEG) signals were acquired from 18 electrodes (F7, F3, Fz, F4, F8, C3, Cz, C4, T5, T3, T4, T6, P3, Pz, P4, O1, Oz, and O2) placed using a 10/20 extended Quik-Cap system (Compumedics Neuroscan, Inc., El Paso, TX). Horizontal and vertical electrooculogram (EOG) activity for eye movements was monitored bipolarly with ocular electrodes placed on the supero-lateral right canthus and infero-lateral to the left eye. A ground electrode was placed on the mid-forehead on the Quik-Cap. References were placed at vertex by default, but were subsequently re-referenced off-line to averaged mastoids. The values of interelectrode impedance were kept at 5 KΩ for all electrodes. The raw EEG signal was recorded with an A/D rate of 500 Hz/channel, a band-pass filter of 0.1–50 Hz, and a 60-Hz notch filter using an on-line amplifier. These data were written continuously to hard disk for off-line analysis using Neuroscan Scan 4.3 analysis software (Compumedics Neuroscan, Inc., El Paso, USA).

Trials with a response error or EEG artifacts (e.g., VEOG, HEOG, and electromyogram) exceeding peak-to-peak deflections over 100 μV were discarded before averaging. ERPs were extracted off-line and averaged in epochs starting 200 ms prior to stimulus activity onset, and lasting for 1,000 ms of post-stimulus activity. Since the ERP P3 component is widely examined among studies that independently and simultaneously investigate the externalizing spectrum and executive functioning (Baskin-Sommers et al., 2014), it was the focus of the current work. The effects of the two cognitive tasks on the P3 component in the elderly were clearly visible in the frontal-parietal regions of the scalp in the current study (see also Kieffaber and Hetrick, 2005; Friedman et al., 2008; Baskin-Sommers et al., 2014). Three electrodes (Fz, Cz, Pz) were thus analyzed in the present work (Themanson et al., 2006; Tusch et al., 2016). P3 mean amplitudes were calculated for the time-window between 300 and 600 ms post stimulus.

### Data processing and statistical analyses

One-way analysis of variance (ANOVA) was used to examine the homogeneity of the demographic backgrounds of the participants at the baseline in the open-skill, closed-skill, and control groups. A two-tailed paired *t*-test was used to analyze the differences within the three groups at baseline and at 24 weeks.

Different trials/conditions in the two cognitive tasks were subjected to neuropsychological (i.e., AR and RT) and electrophysiological (i.e., P3 amplitude) statistical analyses. Only the RT and ERP data corresponding to correct answers were averaged according to the task trial/condition. With regard to the task-switching paradigm, two switch costs were determined by the RTs performance: (1) general-switch cost, which was obtained by subtracting the mean RT between homogeneous trials during homogeneous blocks and non-switch trials during heterogeneous blocks; (2) specific-switch cost, which was calculated by subtracting the mean RT between non-switch trials and switch trials during heterogeneous blocks.

#### Task switching paradigm

With regard to the neuropsychological performance, the AR and RT were separately submitted to a 3 (*Group*: open-skill vs. closed-skill vs. control) × 2 (*Time*: pre-exercise vs. post-exercise) × 3 [*Trial*: homogeneous (during homogeneous blocks) vs. non-switch vs. switch (during heterogeneous blocks)] mixed repeated measures analysis of variance (RM–ANOVA). In terms of the electrophysiological performance, the P3 latency and amplitude from ERP recordings were submitted to a 3 (*Group*: open-skill vs. closed-skill vs. control) × 2 (*Time*: pre-exercise vs. post-exercise) × 3 (*Trial*: homogeneous vs. non-switch vs. switch) × 3 (*Electrode*: Fz vs. Cz vs. Pz) RM–ANOVA.

#### N-back task

With regard to the neuropsychological performance, the AR and RT were separately submitted to a 3 (*Group*: open-skill vs. closed-skill vs. control) × 2 (*Time*: pre-exercise vs. post-exercise) × 3 (*Condition*: 0-back vs. 1-back vs. 2-back) RM–ANOVA. In terms of the electrophysiological performance, the P3 latency and amplitude from ERP recordings were submitted to a 3 (*Group*: open-skill vs. closed-skill vs. control) × 2 (*Time*: pre-exercise vs. post-exercise) × 3 (*Condition*: 0-back vs. 1-back vs. 2-back) × 3 (*Electrode*: Fz vs. Cz vs. Pz) RM–ANOVA.

Bonferroni *post-hoc* analyses were performed when there was a significant difference. Simple main effects were determined following observation of any significant interaction effects. Because cardiorespiratory fitness, social participation, and BMI are confounding factors on cognitive performance (Messier and Gagnon, 2009; Miller et al., 2012), the neuropsychological and electrophysiological performances of the three groups after the exercise intervention were assessed separately using an analysis of covariance (ANCOVA) to examine the effects on the neurocognitive performance in relation to different types of intervention, with the three post-exercise factors as a covariate. The Greenhouse–Geisser (G–G) correction adjusted the significance levels of the *F* ratios whenever RM–ANOVA detected a major violation of the sphericity assumption. Partial Eta squared (η_*p*_^2^) was used to calculate effect sizes for significant main effects and interactions, with the following criteria used to determine the magnitude of mean effect size: 0.01–0.059 indicated a small effect size; 0.06–0.139, a medium effect size; and >0.14, a large effect size. A *p*-value less than 0.05 for the differences between the mean values is considered statistically significant.

## Results

### Demographic characteristics

Table 1 presents an overview of the pre- and post-exercise characteristics of the participants. At baseline there were no significant differences (all *p*s > 0.05) at the group level with regard to age, height, weight, BMI, systolic and diastolic pressure in the three groups (i.e., open-skill, closed-skill, and control). Other confounding factors (e.g., the years of education, social participation, memory depth, global cognitive function, depression, and cardiorespiratory fitness) in relation to cognition and the tests of functional fitness also did not achieve significant difference in the three groups (all *p*s > 0.05) across the three groups at baseline. Paired *t*-tests showed that after the exercise intervention the open-skill group had significantly improved scores for the social participation, arm curl, chair stand, and 8-foot up-and-go items, and approached significance for the level of cardiorespiratory fitness (VO_2max_); and the results for the closed-skill group showed that the values of weight and BMI decreased significantly, the level of cardiorespiratory fitness was significantly enhanced, and the performance of memory depth approached significance.

### Accuracy rate (AR)

#### Task-switching paradigm

**Figure 2:**
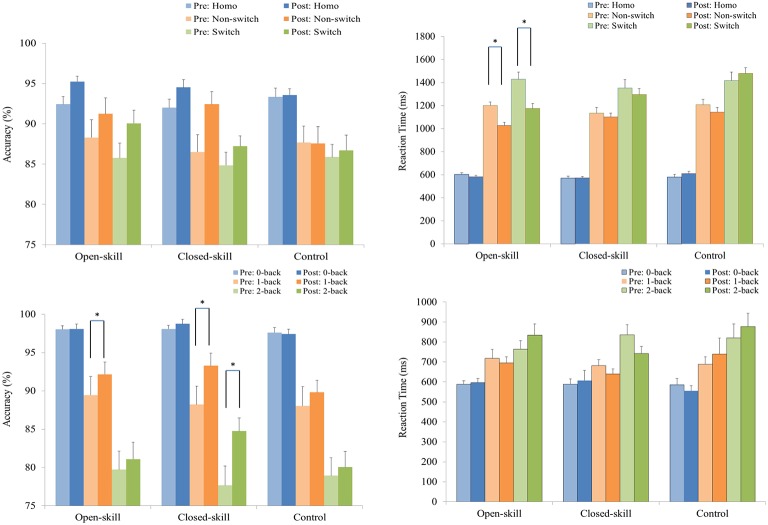
Grand mean accuracy rate (the left side) and reaction time (the right side) performance (mean ± SE) of the open-skill, closed-skill, and control groups during the task-switching (**upper**) and N-back (**lower**) tasks before and after the exercise intervention. ^*^Significantly different (*p* < 0.05).

As shown in Figure 2, RM–ANOVA performed on the ARs only highlighted the main effects of *Time* [*F*_(1, 61)_ = 10.36, *p* = 0.002, η_*p*_^2^ = 0.15] and *Trial* [*F*_(2, 122)_ = 35.57, *p* < 0.001, η_*p*_^2^ = 0.37], with a higher AR post- (90.96%) than pre-exercise (88.53%) and with the following trial gradient across the three groups: homogeneous (93.52%) > non-switch (88.96%) > switch (86.75%).

#### N-back task

RM–ANOVA performed on the ARs for the N-back task revealed the main effects of *Time* [*F*_(1, 61)_ = 20.45, *p* < 0.001, η_*p*_^2^ = 0.25] and *Condition* [*F*_(2, 122)_ = 129.89, *p* < 0.001, η_*p*_^2^ = 0.68], with a higher AR for post- (90.61%) than pre-exercise (88.43%), and with the following condition gradient: 0-back (98.01%) > 1-back (90.17%) > 2-back (80.37%). The interactions between *Time* × *Group* [*F*_(2, 61)_ = 4.74, *p* = 0.012, η_*p*_^2^ = 0.14], *Time* × *Condition* [*F*_(2, 122)_ = 10.51, *p* < 0.001, η_*p*_^2^ = 0.15], and *Time* × *Group* × *Condition* [*F*_(4, 122)_ = 2.61, *p* = 0.012, η_*p*_^2^ = 0.39] were also significant. *Post-hoc* analysis for the *Time* × *Group* × *Condition* interaction showed that (1) there were no significant differences at any condition among the three groups at baseline and after the exercise intervention (all *p*s > 0.05); and (2) when post- compared to pre-exercise, the open- [*F*_(1, 21)_ = 4.44, *p* = 0.047] and closed-skill [*F*_(1, 20)_ = 21.45, *p* < 0.001] groups exhibited significantly higher ARs in the 1-back condition, and only the closed-skill [*F*_(1, 20)_ = 36.59, *p* < 0.001] group exhibited a significantly higher AR in the 2-back condition. In addition, the significant post- and pre-exercise differences in ARs among the three groups only emerged in the 2-back condition, with a greater improvement after the exercise intervention for the closed-skill group than the open-skill and control groups (closed-skill vs. open-skill: *p* = 0.002; closed-skill vs. control: *p* = 0.001). Even when the post-exercise cardiorespiratory fitness, social participation, and BMI were controlled for, the results of ANCOVA on the ARs in the 2-back condition still indicated a significant difference in the three groups [*F*_(2, 58)_ = 9.90, *p* < 0.001], with *post-hoc* analysis indicating that closed-skill group performed significantly better than the open-skill (closed-skill vs. open-skill: *p* < 0.001) and control (closed-skill vs. control: *p* < 0.001) groups after the exercise intervention.

### Reaction time (RT)

#### Task-switching paradigm

As illustrated in Figure 2, RM–ANOVA conducted on mean RTs revealed the main effects of *Time* [*F*_(1, 61)_ = 10.22, *p* = 0.002, η_*p*_^2^ = 0.14] and *Trial* [*F*_(2, 122)_ = 490.16, *p* < 0.001, η_*p*_^2^ = 0.89], suggesting that RTs were faster post- (999.2 ms) rather than pre-exercise (1,056.1 ms), and that RTs followed the trial gradient: homogeneous (587.4 ms) < non-switch (1,136.6 ms) < switch (1,358.9 ms). The interactions of *Time* × *Group* [*F*_(2, 61)_ = 7.34, *p* = 0.001, η_*p*_^2^ = 0.19], *Time* × *Condition* [*F*_(2, 122)_ = 5.55, *p* = 0.005, η_*p*_^2^ = 0.08], and *Time* × *Condition* × *Group* [*F*_(4, 122)_ = 3.69, *p* = 0.007, η_*p*_^2^ = 0.11] were also significant. *Post-hoc* analysis for the *Time* × *Condition* × *Group* interaction showed that (1) the open- and closed-skill groups showed significantly faster responses than the control group in the switch trials after the exercise intervention [*F*_(2, 61)_ = 10.31, *p* < 0.001; open-skill vs. control: *p* < 0.001; closed-skill vs. control: *p* = 0.027], and (2) when post- compared to pre-exercise, the open-skill group responded faster in the non-switch [*F*_(1, 21)_ = 24.80, *p* < 0.001] and switch [*F*_(1, 21)_ = 27.15, *p* < 0.001] trials. Even when the post-exercise cardiorespiratory fitness, social participation, and BMI were controlled for, the results of ANCOVA on the RTs in switch trials still indicated a significant difference in the three groups [*F*_(2, 58)_ = 7.70, *p* = 0.001], with *post-hoc* analysis indicating that the two exercise groups showed significantly faster responses than the control group (open-skill vs. control: *p* < 0.001; closed-skill vs. control: *p* = 0.027) after the exercise intervention.

In terms of RT switch costs, RM-ANOVA for the general-switch cost revealed the main effect of *Time* [*F*_(1, 61)_ = 15.01, *p* < 0.001, η_*p*_^2^ = 0.20], indicating that the general-switch cost was smaller post- (501.9 ms) rather than pre-exercise (596.5 ms) across the three groups. RM-ANOVA for the specific-switch cost revealed that the interaction of *Time* × *Group* [*F*_(2, 61)_ = 3.35, *p* = 0.042, η_*p*_^2^ = 0.10] was significant. *Post-hoc* analysis showed that only the value of the specific-switch cost approached significance [*F*_(1, 21)_ = 4.33, *p* = 0.050] post-exercise (149.2 ± 163.0 ms) relative to pre-exercise (227.5 ± 208.1 ms) in the open-skill group.

#### N-back task

RM–ANOVA conducted on mean RTs for the N-back task only revealed a main effect of *Condition* [*F*_(2, 92)_ = 210.94, *p* < 0.001, η_*p*_^2^ = 0.82], with the following gradient: 0-back (586.8 ms) < 1-back (693.5 ms) < 2-back (812.4 ms).

### P3 latency

#### Task-switching paradigm

**Figure 3:**
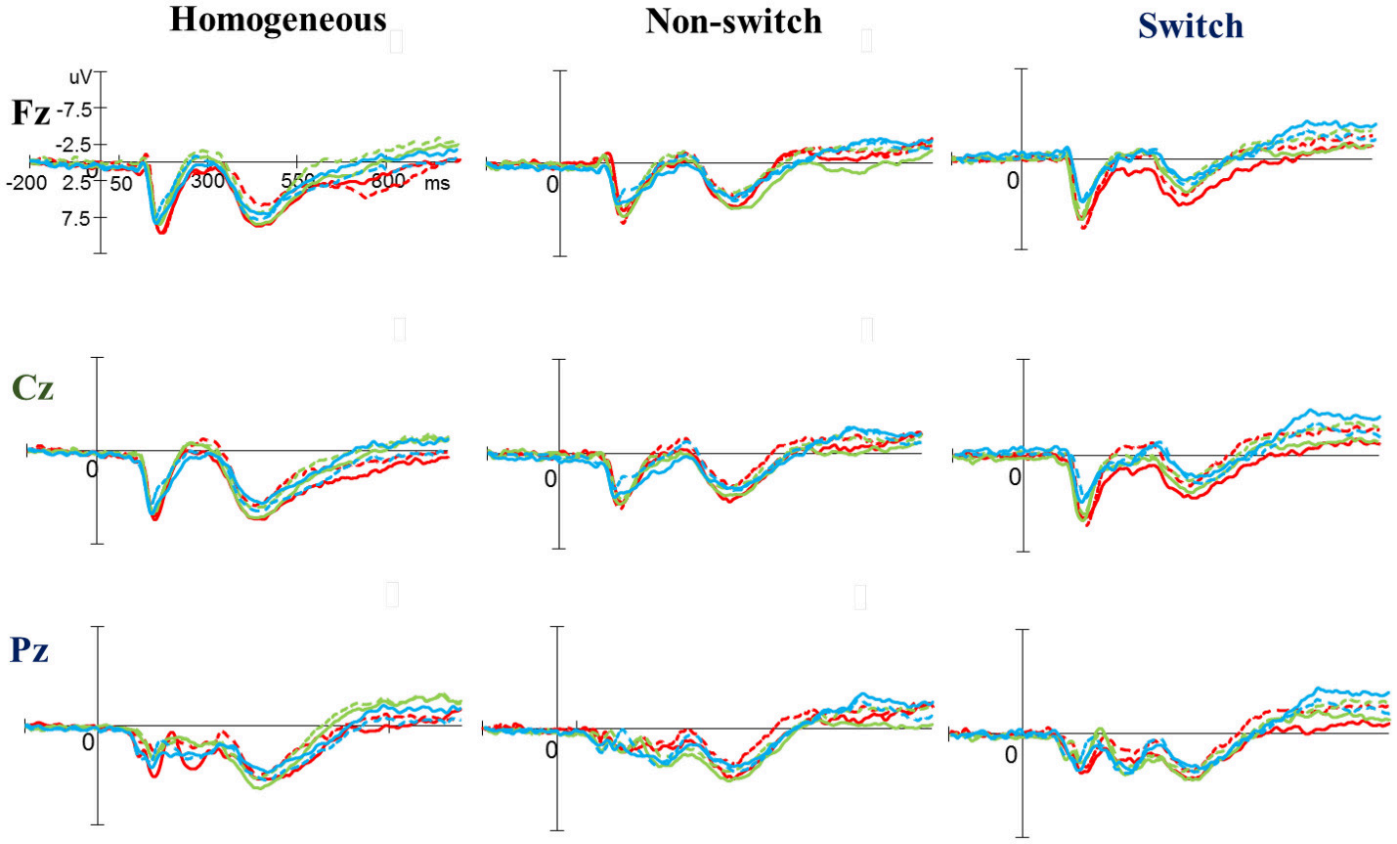
Grand average event-related potential waveforms of three electrodes (Fz, Cz, and Pz) for three conditions (homogeneous, non-switch, and switch) for the P3 component in the task-switching paradigm at baseline (dashed line) and after exercise intervention (solid line) in the open-skill (red), closed-skill (green), and control groups (blue).

As shown in Figures 3, 5, no significant difference was observed with regard to any main effect or interaction in the P3 latency. These results indicate that the P3 latencies did not show obvious changes between three groups when performing the task-switching paradigm throughout the exercise intervention stage.

#### N-back task

No significant difference was observed with regard to any main effect or interaction in the P3 latency. These results indicate that the P3 latencies did not show obvious changes when performing the N-back task between three groups throughout the exercise intervention stage.

### P3 amplitude

#### Task-switching paradigm

As shown in Figures 3, 5, RM–ANOVA performed on the P3 amplitudes of the task-switching paradigm showed main effects of *Time* [*F*_(1, 61)_ = 30.30, *p* < 0.001, η_*p*_^2^ = 0.33], *Condition* [*F*_(2, 122)_ = 39.35, *p* < 0.001, $\eta_{p}^{2}$ = 0.39] and *Electrode* [*F*_(2, 122)_ = 15.99, *p* < 0.001, $\eta_{p}^{2}=0.21$], with the *post-hoc* analyses indicating that the P3 amplitudes were larger post- (4.89 μV) rather than pre-exercise (3.82 μV); the P3 amplitudes in the three conditions had the following gradient: homogeneous (5.85 μV) > non-switching (3.99 μV) > switching (3.22 μV); and significantly greater amplitudes at the Fz (4.73 μV) and Cz (4.57 μV) electrodes were found as compared to the Pz (3.77 μV) electrode. The interactions of *Time* × *Group* [*F*_(2, 61)_ = 11.47, *p* < 0.001, η_*p*_^2^ = 0.27] and *Condition* × *Electrode* [*F*_(4, 244)_ = 13.96, *p* < 0.001, η_*p*_^2^ = 0.19] were also significant. *Post-hoc* analysis for the *Time* × *Group* interaction showed that (1) there were no significant differences among the three groups at baseline [*F*_(2, 61)_ = 0.04; *p* = 0.961]; (2) there were significant differences among the three groups after the exercise intervention [*F*_(2, 61)_ = 6.37; *p* = 0.003], with *post-hoc* analysis showing that both open- (5.79 μV) and closed-skill (5.38 μV) groups showed significantly larger P3 amplitudes as compared to the control group (3.50 μV) after the intervention (open-skill vs. control: *p* = 0.004; closed-skill vs. control: *p* = 0.025); and (3) both the open- and closed-skill groups exhibited significantly larger P3 amplitudes [open-skill: *F*_(1, 21)_ = 32.99, *p* < 0.001; closed-skill: *F*_(1, 20)_ = 23.34, *p* < 0.001] across all three conditions and electrodes after the intervention relative to baseline. Even when the post-exercise cardiorespiratory fitness, social participation, and BMI were controlled for, the results of ANCOVA on the averaged P3 amplitudes in the three conditions and three electrodes still indicated significant post-exercise group differences [*F*_(2, 58)_ = 8.07, *p* = 0.001], with *post-hoc* analysis indicating that the two exercise groups showed significantly larger P3 amplitudes than the control group (open-skill vs. control: *p* < 0.001; closed-skill vs. control: *p* = 0.003) after the intervention.

#### N-back task

**Figure 4:**
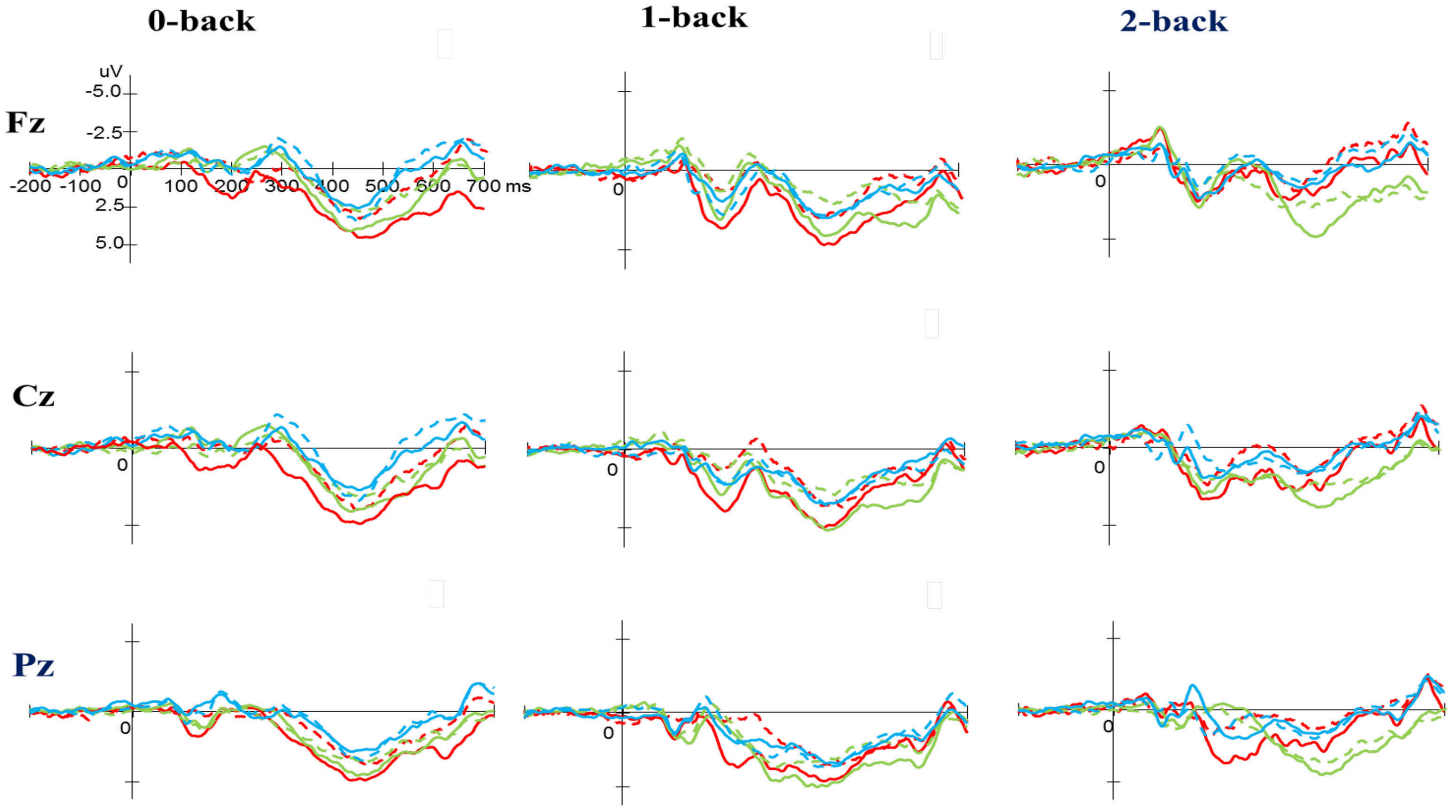
Grand average event-related potential waveforms of three electrodes (Fz, Cz, and Pz) for three conditions (0-, 1-, and 2-back) for the P3 component in the N-back task at baseline (dashed line) and after exercise intervention (solid line) in the open-skill (red), closed-skill (green), and control groups (blue).

As illustrated in Figures 4, 6, RM–ANOVA performed on the P3 amplitudes of the N-back task showed the main effects of *Time* [*F*_(1, 61)_ = 13.42, *p* = 0.001, η_*p*_^2^ = 0.18] and *Condition* [*F*_(2, 122)_ = 9.85, *p* < 0.001, η_*p*_^2^ = 0.14], with the *post-hoc* analyses indicating the P3 amplitudes were larger post- (3.57 μV) rather than pre-exercise (2.27 μV); and significantly greater amplitudes at the 0-back (3.31 μV) and 1-back (3.27 μV) conditions were found as compared to the 2-back (2.19 μV) condition. The interaction of *Time* × *Group* [*F*_(2, 61)_ = 3.89, *p* = 0.026, η_*p*_^2^ = 0.11] was also significant, with *post-hoc* analysis indicating that (1) there were no significant differences among the three groups at baseline [*F*_(2, 61)_ = 0.02; *p* = 0.985]; (2) there were significant differences among the three groups after the exercise intervention [*F*_(2, 61)_ = 6.09; *p* = 0.004], with *post-hoc* analysis showing that both open- (4.02 μV) and closed-skill (4.52 μV) groups showed significantly larger P3 amplitudes as compared to the control group (2.17 μV) after the intervention (open-skill vs. control: *p* = 0.032; closed-skill vs. control: *p* = 0.005); and (3) both the open- and closed-skill groups exhibited significantly larger P3 amplitudes [open-skill: *F*_(1, 21)_ = 7.59, *p* = 0.012; closed-skill: *F*_(1, 20)_ = 16.83, *p* = 0.001] across all three conditions and electrodes after the intervention relative to baseline. Even when the post-exercise cardiorespiratory fitness, social participation, and BMI were controlled for, the results of ANCOVA on the averaged P3 amplitudes in the three conditions and three electrodes still indicated significant post-exercise group differences [*F*_(2, 58)_ = 5.56, *p* = 0.006], with *post-hoc* analysis indicating that the two exercise group showed significantly larger P3 amplitudes than the control group (open-skill vs. control: *p* = 0.016; closed-skill vs. control: *p* = 0.002).

**Figure 5:**
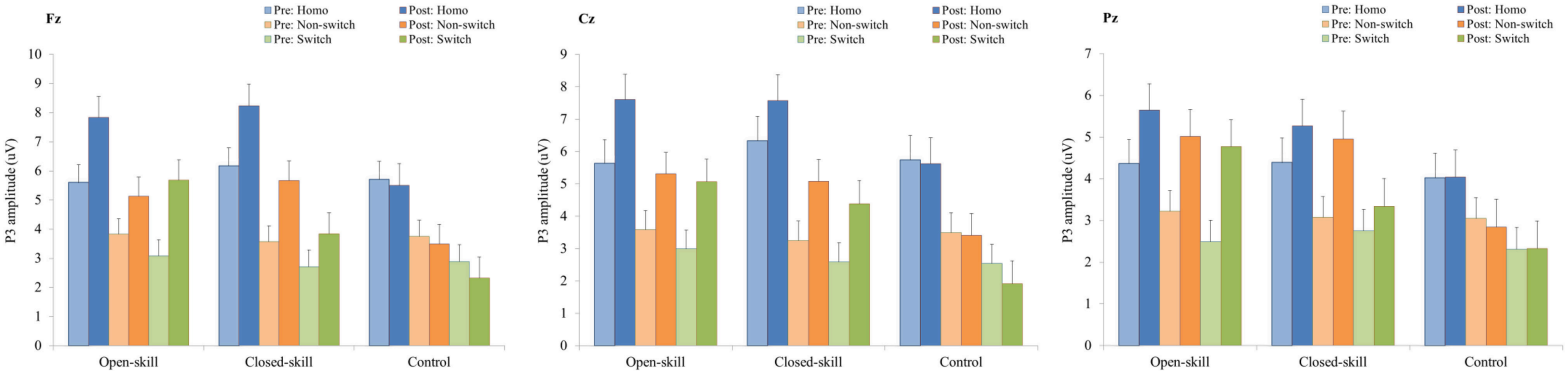
P3 amplitudes (mean ± SE) of three electrodes (Fz, Cz, and Pz) for three conditions (homogeneous, non-switch, and switch) during the task-switching paradigm in the open-skill, closed-skill, and control groups before and after the exercise intervention.

**Figure 6:**
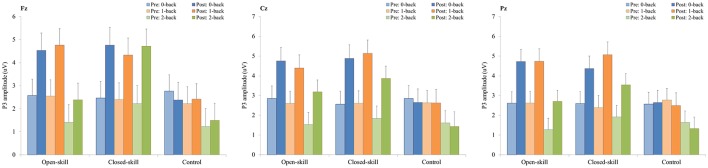
P3 amplitudes (mean ± SE) of three electrodes (Fz, Cz, and Pz) for three conditions (0-, 1-, and 2-back) during the N-back task in the open-skill, closed-skill, and control groups before and after the exercise intervention.

## Discussion

### Main findings

The present study aimed to explore the effects of 6-month open- (e.g., table tennis) and closed-skill (e.g., bike riding or brisk walking/jogging) exercise interventions on neurocognitive performance in the elderly when performing the task-switching paradigm and N-back task. The findings showed that, with regard to the neuropsychological performance, after participation in the two types of exercise the elderly participants did not increase their ARs in the task-switching paradigm, but showed significantly faster responses than the control group in the switch trials of the heterogeneous condition after the exercise intervention. In addition, compared to the baseline, regular participation in open-skill exercise for 6 months could effectively enhance RTs in the elderly when performing the non-switch and switch trials of the heterogeneous condition, and the specific-switch cost approached significance post- relative to pre-exercise in the open-skill group. In terms of the N-back task, although performing the open- and closed-skill exercise for 6 months did not improve RTs in the elderly when performing such a cognitive task, the ARs in the 1-back condition were significantly enhanced after the exercise intervention in both exercise groups, and the beneficial effects on the 2-back condition only emerged in the closed-skill group. In terms of electrophysiological performance, both open- and closed-skill groups exhibited significantly larger P3 amplitudes across conditions and electrodes after the exercise intervention relative to baseline when performing not only the task-switching paradigm, but also the N-back task. These post-exercise neurocognitive benefits still remained even when the confounding factors (e.g., cardiorespiratory fitness, social participation, and BMI) were controlled for.

### Neuropsychological performances

In the present study, the older adults participating in the open-skill types of exercise (e.g., table tennis) not only had to follow the rules of the game, but also switch strategies and select relevant sensory information when encountering the various skill levels of the other players within a constantly changing environment (Di Russo et al., 2010). In addition, they needed to continually adapt or switch to more suitable movements/responses to initiate appropriate actions or inhibit inappropriate ones based on the opponent's actions. The capabilities of motoric and cognitive switching are thus facilitated during this type of exercise. Indeed, with regard to neuropsychological performance in the task-switching paradigm, although no differences in ARs were found among the three groups after the exercise intervention in the present study, only the open-skill group showed faster responses in the non-switch and switch trials of the heterogeneous condition and a lower specific-switch cost after participation in 6-month table tennis exercise training. This result is in line with the previous findings of cross-sectional research (Tsai and Wang, 2015). Since task switching involves stimulus perception and identification, attentional reallocation, task-set updating, response conflict detection, and monitoring processing (Friedman et al., 2008), the elderly subjects participating in the open-skill exercise modes could show greater cognitive flexibility at switching from one task to another. However, both the open and closed-skill groups showed significantly faster responses when compared to the control group in the switch trials of the heterogeneous condition after the exercise intervention, partly supporting the findings of earlier studies which showed that, relative to the control group, the older adults who regularly participated in physical exercise or regular participation in open- or closed-skill exercise displayed a generalized reduction in the time efficiency of the central processing of cognitive functions when performing a task switching paradigm (Hillman et al., 2006; Themanson et al., 2006; Dai et al., 2013; Tsai and Wang, 2015). In addition, it is worth pointing out that, after engaging in 6-month open- or closed-skill exercise, no improvement in the general-switch cost was found in the older adults in the present study, suggesting that the two exercise modes could not facilitate the process of selecting between and coordinating the two competing tasks (Friedman et al., 2008).

Although the older adults participating in the closed-skill types of exercise (e.g., bike riding and jogging) in the present study stayed in a predictable and stable environment to perform the related exercise at their own pace (Di Russo et al., 2010), and thus they had a lower cognitive load than seen with the open-skill exercise, their cardiorespiratory fitness was significantly enhanced. This was because, compared to the open-skill exercise, repeatedly performing similar movements coupled with continuously higher HR maintenance could much more effectively improve cardiorespiratory fitness. Previous studies have demonstrated that physical exercise interventions aimed at increasing cardiorespiratory fitness are associated with improvements in the neuropsychological (e.g., response accuracy) and electrophysiological (e.g., P3 and CNV components) performances of working memory in preadolescent children (Pesce et al., 2009; Kamijo et al., 2011; Tsai et al., 2014a) and the elderly (Voss et al., 2013). The potential mechanisms could be that such an exercise mode can increase cerebral blood flow (Seifert and Secher, 2011), cerebral structure (Colcombe et al., 2003), and brain-derived neutrophic factors (Seifert et al., 2010), and regulate hippocampal neurogenesis and synaptic plasticity (Erickson et al., 2009). Given that previous studies have linked cardiorespiratory fitness to enhanced memory in the elderly (Kramer et al., 2001; Voss et al., 2013), and aerobic exercise is associated with increased hippocampal size and function (Erickson et al., 2011), it is not surprising that the closed-skill group in the present study participating in the exercise type that had the greatest impact on cardiorespiratory fitness showed improved ARs on the N-back task involving moderate and high working-memory demands, which require continual processing and updating (Bopp and Verhaeghen, 2005), although there were still no significantly different ARs among the three groups post-exercise. Nevertheless, it should be noted that the open-skill group also exhibited significantly higher ARs on the N-back task involving a moderate working-memory demand (i.e., the 1-back condition) after the exercise intervention, suggesting that the potentially neuropsychological benefit derived from the open-skill exercise on the working memory should not be negated in the elderly. One possible explanation for this is that the effect of improved cardiorespiratory fitness approached significance in the elderly participating in the table tennis intervention in the present study. The results on the relation between the N-back task and exercise modes in the present study were partly in line with Hötting et al.'s (2012) findings that cycling training improved learning and recognition scores in an episodic memory test in middle-aged adults, while stretching/coordination training only improved the learning score, and such beneficial effects could be attributed in an increase in cardiovascular fitness. However, it is worth noting that, when the cofounding factors (also including cardiorespiratory fitness) were controlled for in the present study, the beneficial effect on the AR in the 2-back condition still remained for the closed-skill group relative to the open-skill and control groups. Therefore, the potential mechanisms of the effects (e.g., the increases in the hippocampal size/function and the cerebral blood flow) of the closed-skill exercise on the working memory in the elderly are worth exploring in the future (Erickson et al., 2011).

### Electrophysiological indices

P3 latencies did not show obvious changes after 6-month exercise intervention in the two exercise groups, suggesting that the perceptual/central processing could not be facilitated by the open- and closed-skill exercise in the elderly when performing the two cognitive tasks. In line with a previous study reporting a larger P3 amplitude for the active rather than for the sedentary elderly when performing the task switching paradigm (Hillman et al., 2006), the older adults participating in the 6-month open- and closed-skill exercises programs in the present study could effectively increase their P3 amplitudes and so have larger P3 amplitudes across all conditions relative to the control group, suggesting that the two physical exercise modes could facilitate the attentional set that makes it possible to better evaluate the stimulus in either of the two tasks. However, the open- and closed-skill groups exhibited similar benefits on the neural processes at work in processing the current task-switching paradigm. These results are somewhat inconsistent with those of a previous cross-sectional study (Tsai and Wang, 2015), in which the older adults regularly participating in open-skill exercise (e.g., table tennis and badminton) showed a significantly larger P3 amplitude in the switch condition when performing the task-switching paradigm compared to their counterparts participating in closed-skill exercise. The lack of consistency in these results may be attributable to the inherently better task-switching capacity that may encourage some older adults to choose an open-skill exercise mode (Snowden et al., 2011; Tsai and Wang, 2015). The current 6-month exercise intervention study seems to clarify that both open- and closed-skill exercise modes could produce similar electrophysiological benefits across all conditions when older adults perform the task-switching paradigm. However, even though lower P3-and-RT correlation was found in the older individuals (Pfefferbaum et al., 1980), the distinctive effects of open-skill exercise on neuropsychological performance (i.e., better exercise-training-induced effects on specific-switch cost and RTs in the heterogeneous conditions) in the elderly, as mentioned above, cannot be ignored.

Similarly, relative to the open-skill exercise, although a closed-skilled exercise intervention could have more neuropsychological benefits (i.e., significantly increasing ARs under the high load condition) on the elderly when performing the N-back task, the effects of greater P3 amplitudes were comparable in the two exercise groups in the present study. This suggests that not only closed- but also open-skill exercise could facilitate the memory-related neural processing which is involved in categorizing incoming information and updating the context of the working memory (e.g., encoding, rehearsal, recognition, and retrieval), due to the greater efficiency by which cognitive resources are allocated (Duncan-Johnson and Donchin, 1977; Donchin and Coles, 1988; Rugg, 1995). Although there were no significant between-group AR differences post-exercise among the three groups, the increase in exercise-induced P3 amplitudes observed in the two exercise groups after the 6-month interventions, as compared with the figures seen before training, could reveal that they allocated more resources for target classification and evaluation, which might result in higher ARs in the working memory task since larger overall P3 amplitudes during the N-back could be associated with better task performance in older adults (Tusch et al., 2016). However, the P3 amplitude could be influenced by a greater latency jitter of P3 in the high than in the low memory condition (Kok, 2001), and regular exercise could change P3 latency in the older adults (see review, Hillman et al., 2003), the potential response jitter thus needs to be clarified in further investigations, since different conditions in the two cognitive tasks are involved in different cognitive loads in the present study.

It is worth noting that there was no significant interaction of *Time* × *Group*× *Electrode* in the present study, suggesting that 6-month open- and closed-skill exercise interventions induced similar electrophysiological effects (i.e., increased P3 amplitudes) from the frontal to parietal cortices. However, previous studies have suggested that different physical exercises could affect the brain in different ways (Erickson et al., 2011; Voelcker-Rehage et al., 2011; Burrel, 2015). For example, Erickson et al. (2011) found that 1-year of aerobic exercise training could effectively increase the size of the anterior hippocampus in the elderly, but not the caudate nucleus and thalamus volumes, accompanied by improved memory function. Moreover, such effects were not shown in the individuals performing a stretching and toning program. Voelcker-Rehage et al.'s (2011) longitudinal research reported that although the older adults participating in cardiorespiratory training aimed at enhancing cardiorespiratory fitness or coordination training to increase fine- and gross-motor body coordination could improve their executive functioning and perceptual speed, although with different effects on speed and accuracy, the two types of exercise had different impacts on neural activity, with an increased activation of the sensorimotor network and less prefrontal activation in the cardiovascular-training group, and increased activation in the visual-spatial network (e.g., right inferior frontal gyrus, superior parietal cortex, thalamus, and caudate body) in the coordination-training group. In addition, animal studies showed that regular cardiovascular training in rats did not increase the number of synapses, but could increase the density of capillaries (Black et al., 1990) and shorten the diffusion distance from the blood vessels in the molecular layer of the paramedian lobule (Isaacs et al., 1992). In contrast, regular complex motor-skill training in the rats did not increase the density of capillaries, but could substantially increase the number of synapses per Purkinje neuron and blood vessels, thus maintaining the diffusion distance (Isaacs et al., 1992). These findings from both human and animal studies suggest that different types of exercise intervention (e.g., open- vs. closed-skill) could produce distinct training effects on the brain tissues and neural activations. However, in the present study the effects of exercise interventions on neural activity in the frontal-to-parietal cortices seem to be comprehensively covered. In fact, in terms of aging, cognition, and brain function, the phenomenon of dedifferentiation, which characterizes a simple marker of cognitive decline, is often found in the elderly. That is, the regions of the brain that are recruited to perform a variety of cognitive tasks are less specific among older rather than younger adults (Cabeza, 2001), possibly due to additional cortical areas being recruited to compensate for losses in neural efficiency in the former. Therefore, while increased prefrontal activation to compensate for processing impairment, particularly in posterior areas, is a consequence of age-related structural and functional declines in various brain regions (Greenwood, 2007), the findings of the present study suggest that both open- and closed-skill exercise modes could not only facilitate anterior cortical processing efficiency, but also compensate for neural processing impairment in the posterior cortical areas due to cognitive aging. In addition, whether the improvement in cognitive function that results from the open- and closed-skill exercise would produce more distinctive effects in different age groups is one area for future works to address. It is likely that longitudinal assessments of the effects of open- and closed exercise interventions on electrophysiological performance in young adults would help clarify the benefits of different types of exercise with regard to cognitive functions.

Colcombe and Kramer's (2003) meta-analytic study suggested that aerobic fitness training increases cognitive performance by 0.5 SD on average, regardless of the exercise training method and types of cognitive task, especially in the executive-control processes. Therefore, although the distinct benefits of open- and closed-skill exercises on both working memory and task switching performance were found in the present research, cardiorespiratory fitness could play an important role and have a beneficial influence on these two types of cognitive functions in older adults (Netz et al., 2011; Voelcker-Rehage et al., 2011; Wang et al., 2016). Additionally, in light of evidence that depression, education, social stimulation, and BMI could also mediate the exercise-cognition association in the elderly (Miller et al., 2012; Ronan et al., 2016; Tomioka et al., 2016), adequate controls to take into account the confounding factors that participants in the intervention groups are impacted by, in addition to physical exercise, need to be considered. Indeed, in the present study the two groups showed different levels of improvements in cardiorespiratory fitness, social participation, and BMI after participation in either open- or closed-skilled exercise. However, when including these improved confounding factors as covariates in the analysis of the improved neurocognitive performance after the exercise intervention, the difference among the three groups remained significant, showing that both types of exercise modes do indeed lead to improved neurocognitive performance.

### Strengths and weaknesses

Although a number of confounding factors which could mediate the exercise-cognition association were rigorously controlled for in the current study, some potential limitations of this work need to be addressed. First, since changes in the brain and neurocognitive performance are not always proportional to each other, a decrease in brain size and plasticity that results in cognitive changes is associated with normal aging (Peters, 2006). Further MRI/fMRI studies to explore the changes in the sizes/densities of the brain tissues and in patterns of brain activation would be helpful to understand the complex relationship between different exercise modes and neurocognitive performance, and to determine the exact mechanism of cognitive enhancement in the elderly. Second, some exercise items that do not aim to increase cardiorespiratory fitness are also included in the category of closed-skill exercise, such as resistance exercise and yoga. It would thus be informative in the future to compare the neurocognitive performances of elderly adults on different cognitive tasks using these closed-skill exercise items, in order to more clearly explain the effects of the closed-skill exercise intervention on executive functioning, especially in the memory domains, in this population. Lastly, the ERP recordings in the present study were referred to a linked mastoid reference, and this is not an ideal zero reference, and thus the task-related effect could have been impacted by this, and a potential bias produced (Yao, 2001). However, the adopted reference did not change the topography map of the P300 components (Yang et al., 2017), and the P3 amplitudes decreased significantly with increasing cognitive loads (Kok, 2001) in the present study. These findings suggest that the electrophysiological findings from such a reference site are still reliable. However, further EEG-fMRI studies aiming to explore the effects of exercise interventions might consider applying the Reference Electrode Standardization Technique (REST) (Yao, 2001; Yao et al., 2005) as the reference method for ERP data (Yang et al., 2017).

## Conclusions

Extending earlier cross-sectional studies on aging that relied on volunteer participants, which could inevitably include some selection bias with an overrepresentation of individuals with inherently higher executive functioning, the present study of a 6-month exercise intervention confirmed recent cross-sectional results showing that open- and closed-skill exercise modes relate differently to various forms of executive functioning (e.g., task switching and working memory) in relation to cognitive aging in older adults. However, the current findings more clearly revealed that both open- and closed-skill exercise could effectively enhance overall brain cortical activity. These beneficial effects of the two exercise interventions on the neuropsychological and electrophysiological performances in the elderly remained unchanged after statistical adjustment for improved cardiorespiratory fitness, social participation, and BMI. Although exercise is a simple and healthy lifestyle factor that has been proposed to be protective against neurocognitive declines during aging, possibly even retarding or reversing age-associated degeneration in the brain, different exercise modes seem to have different effects on various forms of executive function in the elderly.

## Author contributions

CT designed the study, wrote the protocol, and the first draft of the manuscript. CP analyzed the data. FC and YT helped collect data.

### Conflict of interest statement

The authors declare that the research was conducted in the absence of any commercial or financial relationships that could be construed as a potential conflict of interest.
